# Duration of Caloric Restriction Modulates Brain Redox Adaptation and Frailty During Aging

**DOI:** 10.3390/ijms27167229

**Published:** 2026-08-13

**Authors:** Desanka Milanovic, Sladjan Pavlović, Milica Prvulovic, Slavica Borković Mitić, Srdjan Sokanovic, Smilja Pracer, Aleksandra Mladenovic

**Affiliations:** 1 Department of Neurobiology, Institute for Biological Research “Sinisa Stankovic”-National Institute of Republic of Serbia, University of Belgrade, Bul. D. Stefana 142, 11060 Belgrade, Serbia; desan@ibiss.bg.ac.rs (D.M.); milica.prvulovic@ibiss.bg.ac.rs (M.P.); srdjan.sokanovic@ibiss.bg.ac.rs (S.S.); smilja.pracer@ibiss.bg.ac.rs (S.P.); 2 Department of Physiology, Institute for Biological Research “Sinisa Stankovic”-National Institute of Republic of Serbia, University of Belgrade, Bul. D. Stefana 142, 11060 Belgrade, Serbia; sladjan@ibiss.bg.ac.rs (S.P.); borkos@ibiss.bg.ac.rs (S.B.M.)

**Keywords:** caloric restriction, frailty index, aging, oxidative stress, antioxidant enzymes, brain, rat

## Abstract

Caloric restriction (CR) is among the most effective nutritional interventions for promoting healthy aging, yet the influence of intervention duration and age at initiation on its efficacy remains incompletely understood. This study investigated the effects of short-term caloric restriction (STCR) and long-term caloric restriction (LTCR) on frailty and brain redox homeostasis in male Wistar rats. Animals were maintained under ad libitum (AL) feeding, or subjected to LTCR initiated at 6 months of age or STCR initiated at 15 or 21 months, and were sacrificed at 18 or 24 months. Frailty, brain oxidative damage, antioxidant defense, systemic redox status, and the protein expression of key redox-related enzymes were evaluated. Both LTCR and STCR improved frailty and enhanced antioxidant defenses in 18-month-old rats. In contrast, in 24-month-old rats STCR was associated with a pronounced glutathione-related antioxidant response and reduced macromolecular oxidation without improving frailty. Systemic redox indices further supported age-dependent metabolic adaptation rather than a uniform antioxidant effect of CR. Overall, these findings demonstrate that CR is associated with age- and duration-dependent differences in redox-related parameters in the brain tissue. The results indicate differential regulation of antioxidant responses under the experimental conditions applied, while the underlying mechanisms and their functional significance require further investigation.

## 1. Introduction

Aging is characterized by a progressive decline in physiological function and increased susceptibility to chronic diseases, particularly cardiovascular, metabolic, and neurodegenerative disorders. However, recent scientific advances have further challenged the view of aging as a gradual linear process. Instead, aging appears to proceed through distinct biological transitions, with accelerated changes reported around the ages of 40–45, 60, and 78 years in humans [1,2,3], accompanied by alterations in metabolism, immune regulation, vascular integrity, and cellular stress responses. Moreover, aging does not occur uniformly across the body. The concept of differential aging recognizes that organs and tissues age at different rates, resulting in considerable heterogeneity both among individuals and between organ systems within the same individual [3,4].

Among the factors influencing aging trajectories, nutrition is increasingly recognized as one of the most important modifiable determinants of biological aging. Dietary patterns affect multiple hallmarks of aging and influence both lifespan and healthspan in experimental animals and humans [5,6,7,8]. These effects are largely mediated by oxidative stress, one of the central mechanisms of aging characterized by an imbalance between reactive oxygen and nitrogen species and antioxidant defenses. In the brain, oxidative stress contributes to processes such as mitochondrial dysfunction, synaptic impairment, protein oxidation, and neuronal loss, thereby promoting cognitive decline and neurodegenerative disease [9,10]. The brain is uniquely susceptible to oxidative damage caused by oxidative stress, because of its immense metabolic demands and its abundance of metals such as iron and copper. When these metals interact with hydrogen peroxide, they trigger the production of highly reactive hydroxyl radicals. This vulnerability is further compounded by the brain’s distinct structural composition. Its neural membranes are rich in polyunsaturated fatty acids that are readily targeted by lipid peroxidation. Ultimately, oxidative stress in central nervous tissue rarely stems from a single source. Instead, it arises from a complex interplay of mitochondrial failure, neuroinflammation and environmental triggers [11].

Caloric restriction (CR), defined as a reduction in energy intake without malnutrition, is among the most extensively studied nutritional interventions for promoting healthy aging. Across a wide range of species, CR improves metabolic flexibility, enhances cellular stress resistance, modulates inflammatory signaling, and attenuates oxidative damage [5,7,12,13]. However, evidence now strongly points to the fact that the effects of CR depend on several factors: degree of restriction, the age at which it is initiated and the duration of the intervention. Long-term CR initiated early in life is generally considered preventive, whereas CR introduced later in life in some cases may slow or partially reverse pre-existing age-related changes. Despite extensive investigation of CR, relatively few studies have directly compared preventive and interventional CR paradigms, particularly with respect to oxidative balance in the aging brain [14,15,16].

Our research group has previously shown that lifelong CR initiated in young adulthood improves synaptic plasticity, neurotrophic signaling, and emotional and motor behavior and reduces frailty in aging rats [17,18,19,20]. In contrast, CR initiated later in life and applied for shorter durations was associated with less pronounced benefits or even led to negative outcomes in frailty and cognitive performance [19,20,21]. More recently, we demonstrated that CR regimens differing in onset and duration induce distinct redox adaptations in the liver and heart, indicating that the biological effects of CR are shaped by both age at initiation and tissue-specific adaptive responses [22]. Given the organ-specific nature of aging and the particularly high metabolic demands and oxidative vulnerability of the brain, it remains unclear whether similar age-dependent redox adaptations occur within neural tissue.

Therefore, the present study was designed as an extension of our previous work on CR-induced redox remodeling in peripheral organs. We compared oxidative stress and antioxidant balance in the brain tissue in late-middle-aged (18-month-old) and aged (24-month-old) male Wistar rats exposed to CR regimens differing in onset and duration. Markers of oxidative damage, including protein carbonyls and lipid peroxidation, were assessed alongside the activities (superoxide dismutase (SOD, catalase (CAT), glutathione peroxidase (GSH-Px), and (glutathione reductase) GR) and protein expression of key antioxidant enzymes (SOD, CAT, inducible nitric oxide synthase (iNOS)). In addition, systemic redox indices (TAS (Total Antioxidant Status), TOS (Total Oxidant Status), and OSI (Oxidative Stress Index)) were analyzed to characterize age-dependent differences and determine whether distinct CR regimens produce different redox phenotypes in the aging brain. In addition, we evaluated general wellbeing by measuring frailty levels in animals, which reflect cumulative physiological decline associated with aging and oxidative-stress-related damage.

## 2. Results

### 2.1. Physiological Characterization of the Experimental Groups

**Figure 1:**
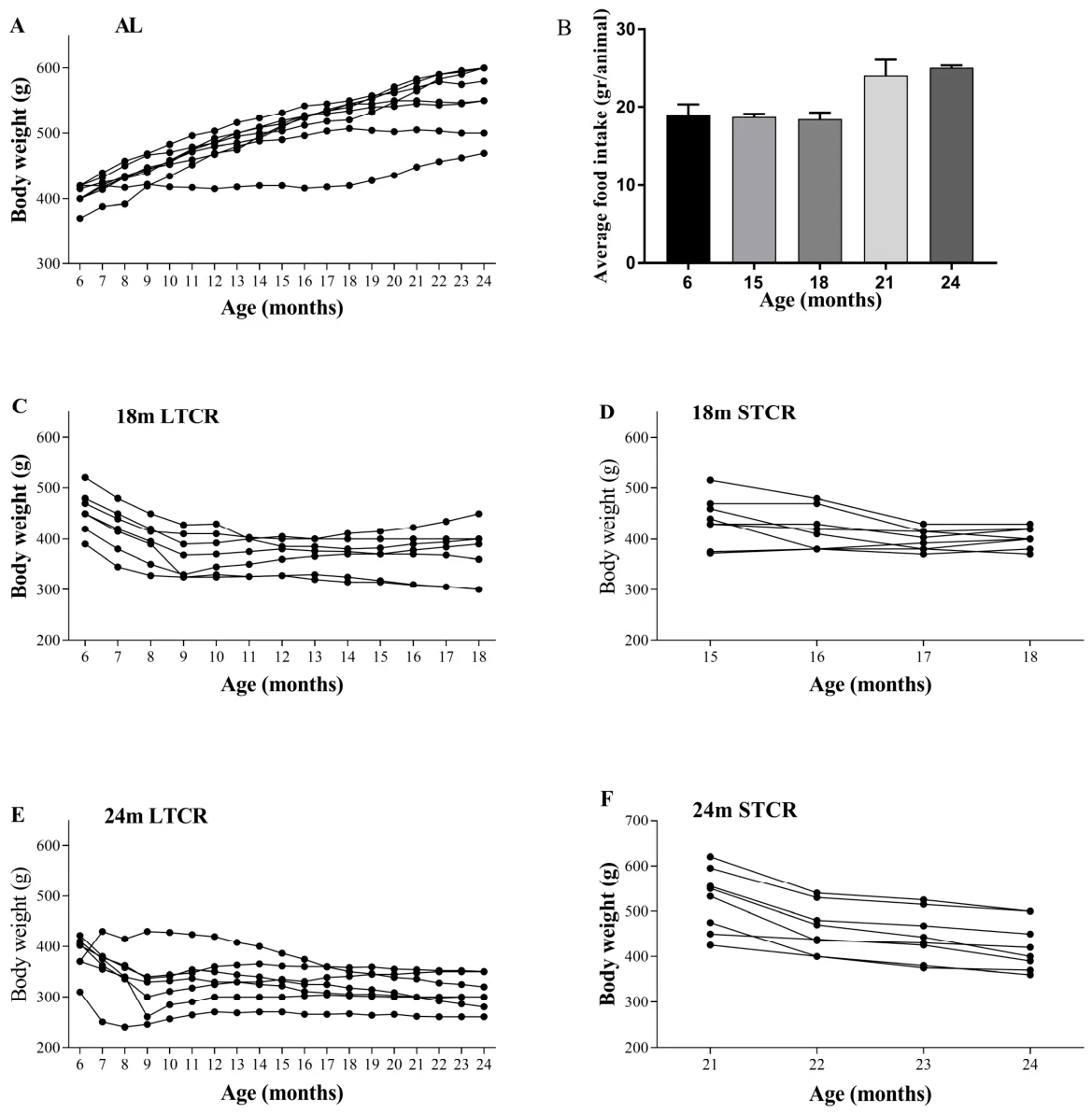
Individual body weight trajectories of male rats in the different dietary groups. (**A**) Body weights of ad libitum (AL)-fed animals. *n* = 8; (**B**) Mean daily food intake of AL-fed animals throughout the experiment. Food intake was calculated based on cage consumption (food provided minus food remaining), with each cage considered as the experimental unit. Data are presented as mean ± SEM calculated from individual cages. (**C**) Body weights of animals subjected to dietary restriction (CR) from 6 to 18 months of age. *n* = 7; (**D**) Body weights of animals subjected to CR from 15 to 18 months of age. *n* = 6; (**E**) Body weights of animals subjected to CR from 6 to 24 months of age. *n* = 7; (**F**) Body weights of animals subjected to CR from 21 to 24 months of age. *n* = 8.

Physiological characterization has been performed based on the changes in the body weight, food intake and frailty level of experimental groups. Food intake and body weight (BW) were monitored weekly and monthly, respectively, throughout the experiment. Control, AL-fed animals exhibited a progressive increase in BW across the study period (Figure 1A). Food intake remained relatively stable between 6 and 18 months of age but increased by approximately 26% from 21 months onward (Figure 1B), coinciding with an acceleration of BW gain. Consequently, 18- and 24-month-old AL-fed animals displayed BW increases of 28% and 37%, respectively, relative to their BW at 6 months of age.

In contrast, animals subjected to long-term CR showed an initial decrease in BW (during the first three months of CR in the LTCR group), followed by stabilization thereafter (Figure 1C,D). As a result, LTCR animals exhibited BW reductions of approximately 18–20% at both 18 and 24 months of age relative to their BW at the onset of CR intervention.

Short-term CR decreased BW in both age groups. Between 15 and 18 months of age we detected a net BW loss of 8% (Figure 1E), while in animals that started CR at 21 months of age, a 19% reduction relative to baseline BW was detected (Figure 1F).

Food intake remained relatively stable during aging and did not differ significantly between 6 and 18 months of age, averaging approximately 20 g per animal per day. However, an increase in food consumption was observed at later ages. Specifically, animals aged 21 and 24 months consumed an average of 24 g and 25 g of food per day, respectively, indicating a moderate rise in food intake during advanced aging.

**Figure 2:**
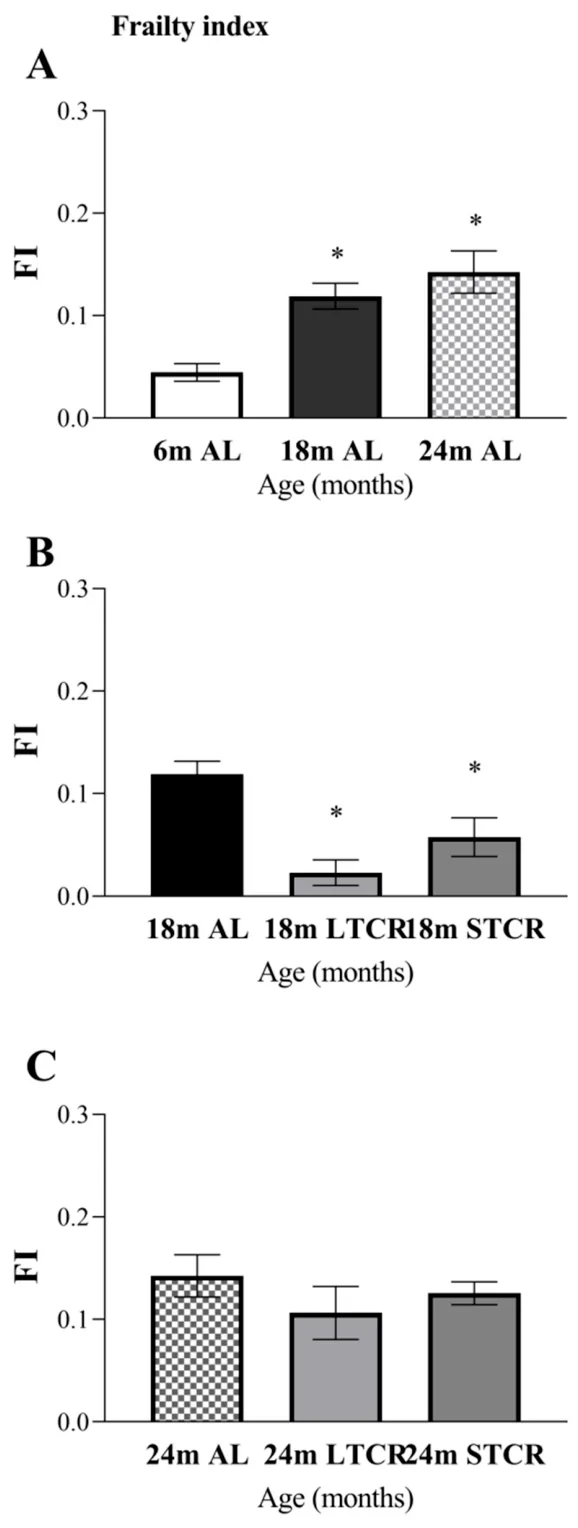
Frailty index in ad-libitum-fed and calorie-restricted male Wistar rats. Frailty index was assessed in AL-fed animals (**A**) and in animals subjected to short- or long-term caloric restriction (CR), sacrificed at 18 (**B**) or 24 (**C**) months of age. Values are presented as mean ± SEM, * *p* < 0.05; *n* (6 m AL) = 11, *n* (18 m AL) = 10*, n* (24 m AL) = 14, *n* (18 m LTCR) = 9, *n* (18 m STCR) = 8, *n* (24 m LTCR) = 8, *n* (24 m STCR) = 9. One-way ANOVA with Dunnett’s post hoc test.

To obtain a more comprehensive assessment of the overall condition of male Wistar rats during aging and under different CR regimens, the frailty index (FI) was determined (Supplementary Table S1). The frailty index quantifies the proportion of age-related health deficits present in an individual, reflecting overall health status and vulnerability to adverse outcomes, and could serve as very useful measure of the effects of caloric restriction [7]. Individual organ systems and physiological parameters were initially evaluated separately (Supplementary Tables S2 and S3), after which the obtained scores were integrated to calculate the FI (Figure 2).

Based on the assessed parameters, the frailty index was calculated for all experimental groups and, as expected, progressively increased with aging under ad libitum feeding conditions (F(2, 30) = 11.23, *p* = 0.0002). Specifically, 18-month-old AL animals exhibited a 2.5-fold increase in frailty index value compared to 6-month-old AL animals (post hoc: *p* = 0.0044), while by the age of 24 months an approximately 3-fold increase was reached (post hoc: *p* = 0.0002) (Figure 2A). Analysis of the obtained scores revealed that among parameters that determine FI some are evidently affected by age more than others. AL aging rats most frequently exhibited impairments in cognitive function, as reflected by the discrimination index (DI), used to assess memory performance, and the SAB (Spontaneous Alternation Behavior) parameter of spatial memory assessment. The next most prevalent age-associated alterations included increased incidences of tumors, rectal prolapse, and poor coat condition, characterized by reduced coat quality, loss of shine, and impaired grooming maintenance (Supplementary Table S2).

Eighteen-month-old rats subjected to CR exhibited significantly lower frailty index values (Figure 2B) compared to their age-matched AL counterparts (F(2, 22) = 10.97, *p* = 0.0005). Early-onset long-term CR reduced frailty index values by approximately fivefold (*p* = 0.0003), whereas late-onset and short-term CR resulted in an approximately twofold reduction (*p* = 0.0123) relative to 18-month-old AL fed animals (Figure 2B). Thus, in 18-month-old animals, both long- and short-term caloric restriction were effective in attenuating age-related changes. The most notable diet–response benefits were observed through the lower incidence of poor coat condition, rectal prolapse, and impaired cognitive performance, compared to age-matched AL controls (Supplementary Table S3).

In contrast, in 24-month-old animals the effectiveness of CR was lesser and non-significant, regardless of duration and onset (Figure 2C). The beneficial effects of reduced food intake were primarily reflected in a lower incidence of tumors, whereas cognitive performance remained similar to that observed in age-matched AL animals (Supplementary Table S3). These findings indicate that the beneficial effects of CR on overall frailty may be more pronounced when the intervention is initiated earlier in the aging process.

### 2.2. SOD and CAT Activities in the Aging Brain Tissue Under Caloric Restriction Conditions

While SOD acts as a primary antioxidant defense in the brain, CAT exhibits relatively low activity in most brain regions compared to other tissues. In the central nervous system, hydrogen peroxide is predominantly cleared by the GSH-Px system, whereas CAT serves as a complementary defense mechanism, primarily localized within peroxisomes [10].

**Figure 3:**
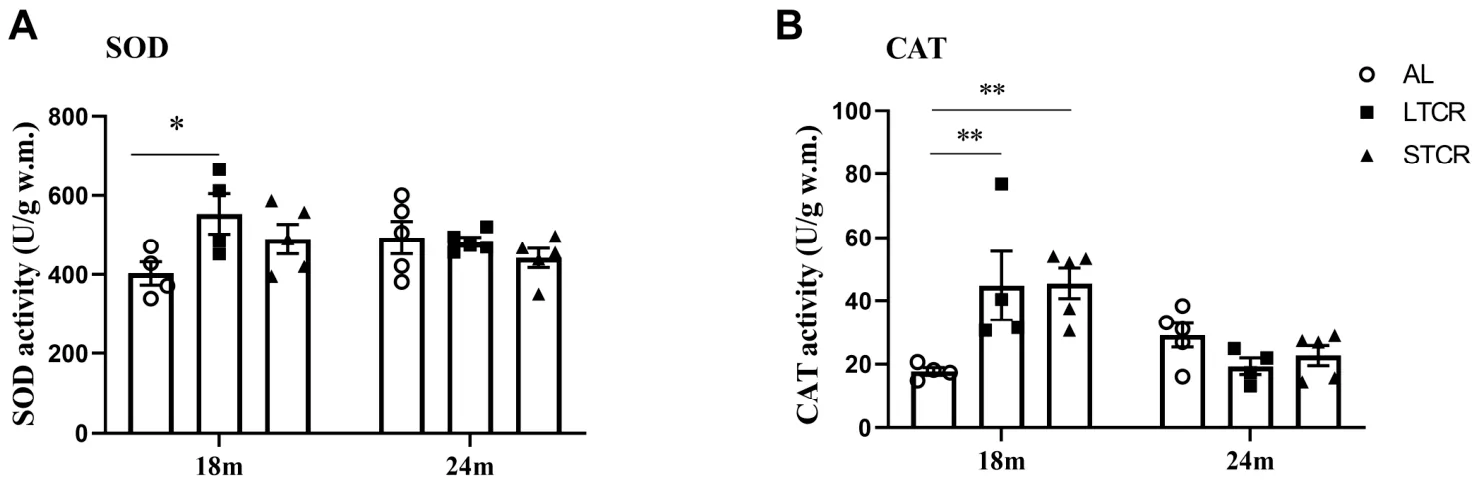
Activity of antioxidant enzymes in the brain of 18- and 24-month-old rats following caloric restriction of different durations. (**A**) Superoxide dismutase (SOD) activity; (**B**) catalase (CAT) activity. AL, ad libitum; STCR, short-term caloric restriction; LTCR, long-term caloric restriction. Values are presented as mean ± SEM. * *p* < 0.05, ** *p* < 0.01. *n* (18 m AL) = 4, *n* (24 m AL) = 5, *n* (18 m LTCR) = 4, *n* (18 m STCR) = 5, *n* (24 m LTCR) = 4, *n* (24 m STCR) = 5. One-way ANOVA with Dunnett’s post hoc test.

We have not detected any significant changes in the activities of those enzymes during normal brain aging. SOD and CAT showed similar activity levels in 18 and 24 m AL groups (Figure 3A,B). However, in 18-month-old animals subjected to LTCR, SOD activity was increased by 37% (*p* < 0.05), whereas the slight increase following STCR was not statistically significant (Figure 3A). In 24-month-old animals, SOD activity did not differ among dietary groups.

CAT activity was significantly increased in 18-month-old animals after both LTCR and STCR, by 2.5-fold compared to age-matched AL controls (*p* < 0.01 for both comparisons). In contrast, in the 24-month-old group, CAT activity showed a slight, but non-significant, decrease after both CR regimens (22–34%) (Figure 3B).

### 2.3. Glutathione-Dependent Antioxidant Enzyme Activities Across Age and Caloric Restriction Conditions

Next, we analyzed the activities of enzymes within the glutathione (GSH)-dependent activities: GSH-Px, GR, and GST (glutathione S-transferase). These enzymes rely on GSH to detoxify ROS (Reactive Oxygen Species) and maintain intracellular redox homeostasis [9].

**Figure 4:**
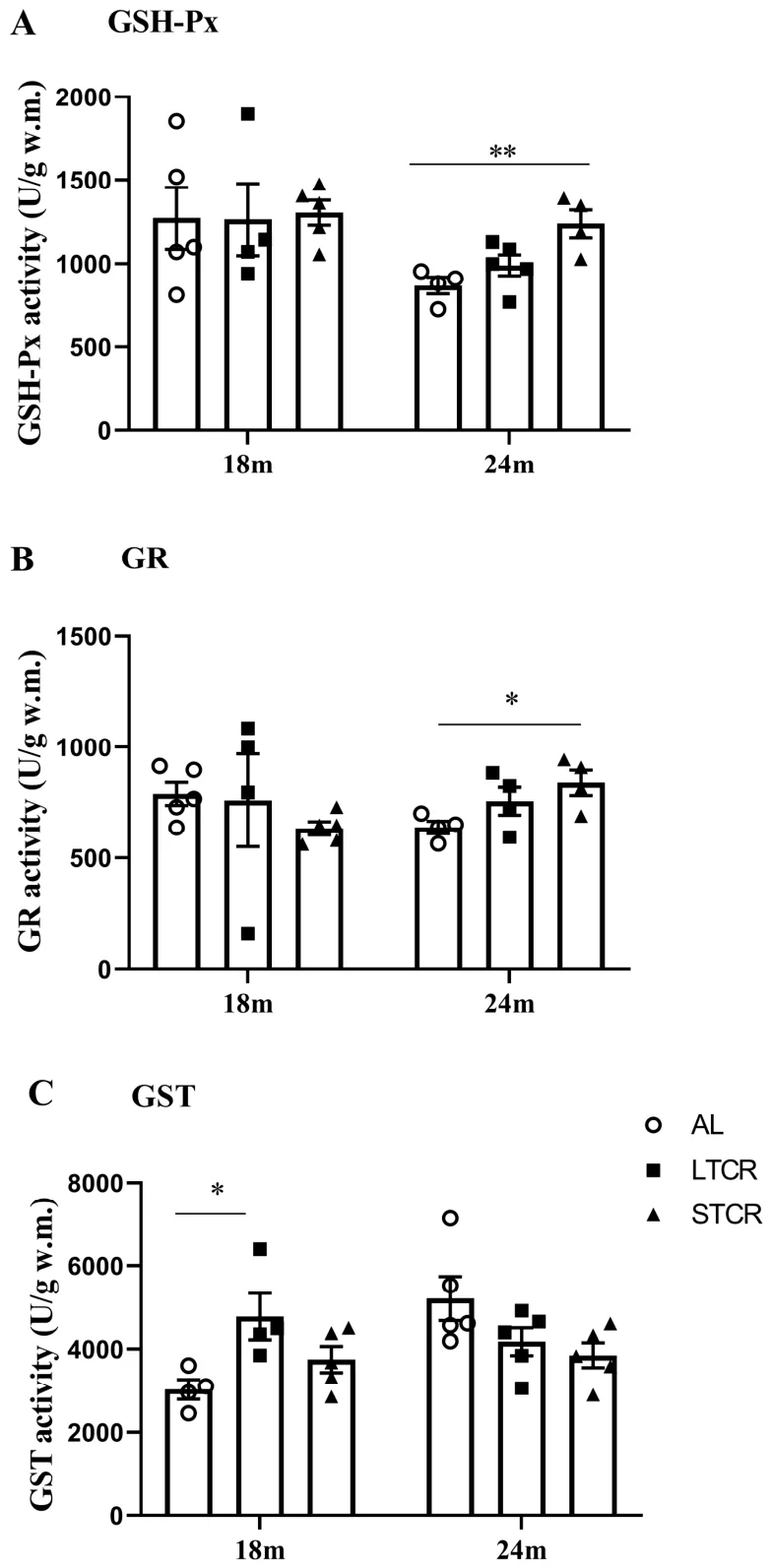
Activity of glutathione-dependent antioxidant enzymes in the brains of 18- and 24-month-old rats following different durations of caloric restriction. (**A**) Glutathione peroxidase (GSH-Px) activity; (**B**) Glutathione reductase (GR) activity; (**C**) Glutathione-S-transferase activity. AL, ad libitum; LTCR, long-term caloric restriction; STCR, short-term caloric restriction. Values are presented as mean ± SEM, * *p* < 0.05, ** *p* < 0.01. *n* (18 m AL) = 4–5, *n* (24 m AL) = 4–5, *n* (18 m LTCR) = 4, *n* (18 m STCR) = 5, *n* (24 m LTCR) = 4–5, *n* (24 m STCR) = 4–5, One-way ANOVA with Dunnett’s post hoc test.

GSH-Px catalyzes the reduction of hydrogen peroxide and lipid peroxides to water and alcohols using reduced glutathione as a cofactor. During aging we detected a decrease in GSH-Px activity, but statistical significance was not reached. Further, its activity did not change under different dietary conditions at 18 months of age, as there were no significant differences among dietary groups in 18-month-old AL and CR animals (Figure 4A). However, in 24-month-old rats, STCR group exhibited a 42% (*p* < 0.01) increase in GSH-Px activity.

GR regenerates reduced GSH from its oxidized form, providing continuous antioxidant protection. GR activity of 18- and 24-month-old AL-fed animals had similar levels. Similarly to GSH-Px, in 18-month-old animals, GR activity was unchanged across dietary groups (Figure 4B), while a statistically significant increase in its activity was detected in the 24-month-old STCR group (31% increase compared to AL, *p* < 0.05). Interestingly, for both enzymes, GSH-Px and GR, a modest increase in the activity was detected in 24-month-old rats after long-term CR, but without statistical significance, demonstrating specific duration/onset-dependent effects of CR.

GST catalyzes the conjugation of GSH with electrophilic compounds, aiding in the detoxification of xenobiotics and reactive by-products. In 18-month-old animals, GST activity was significantly increased only in the LTCR group (57% increase, *p* < 0.05), while in 24-month-old animals (Figure 4C) the slight decline was not statistically significant following any dietary regimen.

### 2.4. Age- and Diet-Dependent Protein Expression of Antioxidant and Redox-Related Enzymes

To determine whether changes in enzyme activity are associated with altered expression at the protein level, we analyzed brain tissue levels of SOD1 and CAT proteins by Western blot. Additionally, inducible nitric oxide synthase (iNOS) protein expression was measured as an indicator of inflammatory nitric oxide production and nitrosative stress. Unlike constitutive NOS isoforms, iNOS activity is largely governed by its expression level, making protein abundance a reliable indicator for its functional output [23].

**Figure 5:**
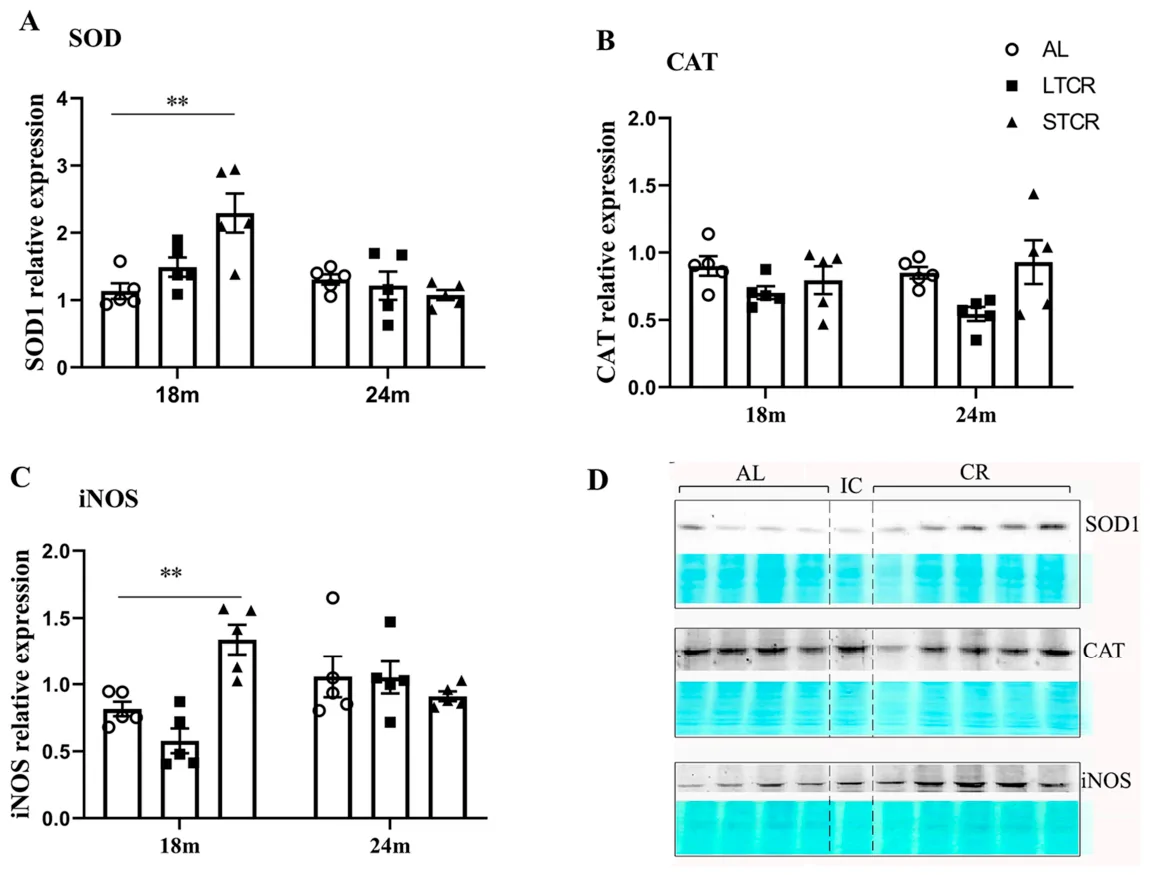
Protein expression of antioxidant and pro-oxidant enzymes in the cortex of 18- and 24-month-old rats following caloric restriction of different durations. (**A**) Superoxide dismutase (SOD) protein level; (**B**) Catalase (CAT) protein level; (**C**) inducible nitric oxide synthase (iNOS) protein level; (**D**) representative immunoblots of SOD1, CAT and iNOS for 18-month-old STCR group. Equal amounts of total protein (15 µg) were loaded per lane. Membranes were stained with Pierce Reversible Protein Stain for loading control and digitally captured using the iBright imaging system. For presentation purposes brightness and contrast were adjusted uniformly across the image, without altering relative band intensities. Linearity of signal with respect to protein loading was verified in preliminary experiments performed under the same experimental conditions, using pooled samples from the study and covering a loading range that includes 15 µg; AL, ad libitum, LTCR, long-term caloric restriction, STCR, short-term caloric restriction, IC, internal control for Western blot quantification. Values are presented as mean ± SEM, ** *p* < 0.01. *n* (18 m AL) = 5, *n* (24 m AL) = 5, *n* (18 m LTCR) = 5, *n* (18 m STCR) = 5, *n* (24 m LTCR) = 5, *n* (24 m STCR) = 5, One-way ANOVA with Dunnett’s post hoc test.

While during physiological aging expression levels of all three proteins remained unchanged, CR changed total protein level in an onset- and duration-dependent manner. In 18-month-old animals SOD1 protein abundance was increased in the LTCR group (31%), although not significantly (Figure 5A). In contrast, the STCR group exhibited a strong 2-fold increase in SOD1 protein abundance (*p* < 0.01) compared to the AL control. In the 24-month-old animals, SOD1 levels remained unchanged in all dietary groups.

CAT protein expression was minimally altered across dietary groups in both 18- and 24-month-old animals (Figure 5B).

iNOS is a redox-related enzyme involved in nitrosative and oxidative stress, particularly in proinflammatory or age-related conditions. In 18-month-old animals, iNOS level was reduced by 29% in the LTCR group compared with AL controls, although this change did not reach statistical significance. Conversely, the STCR group showed significantly higher iNOS level (64% compared with AL group; *p* < 0.01, Figure 5C). No significant differences in iNOS expression were observed in the 24-month-old animals across dietary groups.

### 2.5. Association Between CR and the Lipid and Protein Oxidative Damage

To assess the downstream consequences of altered redox balance, we measured lipid peroxidation and protein carbonyl levels as markers of oxidative damage.

**Figure 6:**
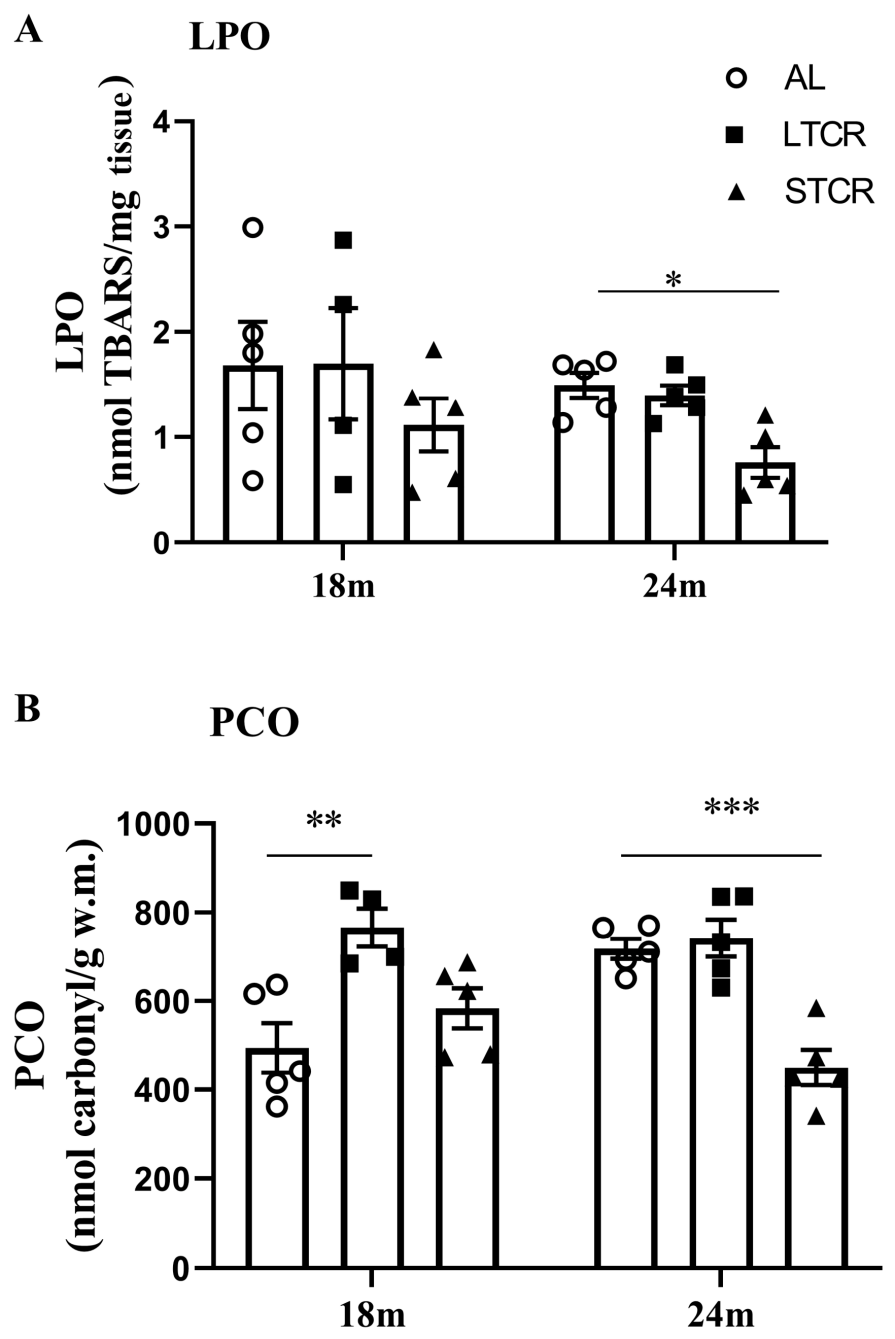
Oxidative modification of lipids and proteins in the brains of 18- and 24-month-old rats following caloric restriction of different durations. (**A**) Lipid peroxidation (LPO) levels; (**B**) protein carbonylation (PCO) levels. AL, ad libitum; LTCR, long-term caloric restriction; STCR, short-term caloric restriction. Values are presented as mean ± SEM, * *p* < 0.05, ** *p* < 0.01, *** *p* < 0.001. *n* (18 m AL) = 5, *n* (24 m AL) = 5, *n* (18 m LTCR) = 4, *n* (18 m STCR) = 5, *n* (24 m LTCR) = 5, *n* (24 m STCR) = 5, One-way ANOVA with Dunnett’s post hoc test.

There were no changes in the LPO (Lipid Peroxidation) levels during aging. Similarly, LPO levels remained unchanged following dietary interventions in 18-month-old animals. However, in 24-month-old animals, STCR was associated with a 49% lower LPO level compared to AL age-matched controls (Figure 6A).

PCO (protein carbonylation) level, by contrast, was significantly influenced by aging as 24-month-old AL animals have 45% (*p* < 0.05) higher protein damage compared to 18-month-old AL-fed animals. Dietary regimes were able to modulate PCO level (Figure 6B): it was significantly higher (by 55%, *p* < 0.01) in 18-month-old animals in the LTCR group and reduced by 37% (*p* < 0.01) in 24-month-old animals after STCR.

### 2.6. Global Redox Status Across Age and Caloric Restriction Conditions

To obtain a more comprehensive assessment of redox status in the examined brain tissue, we measured TAS and TOS and calculated the OSI (TOS/TAS ratio), which together reflect the balance between antioxidant defenses and oxidant burden [24].

**Figure 7:**
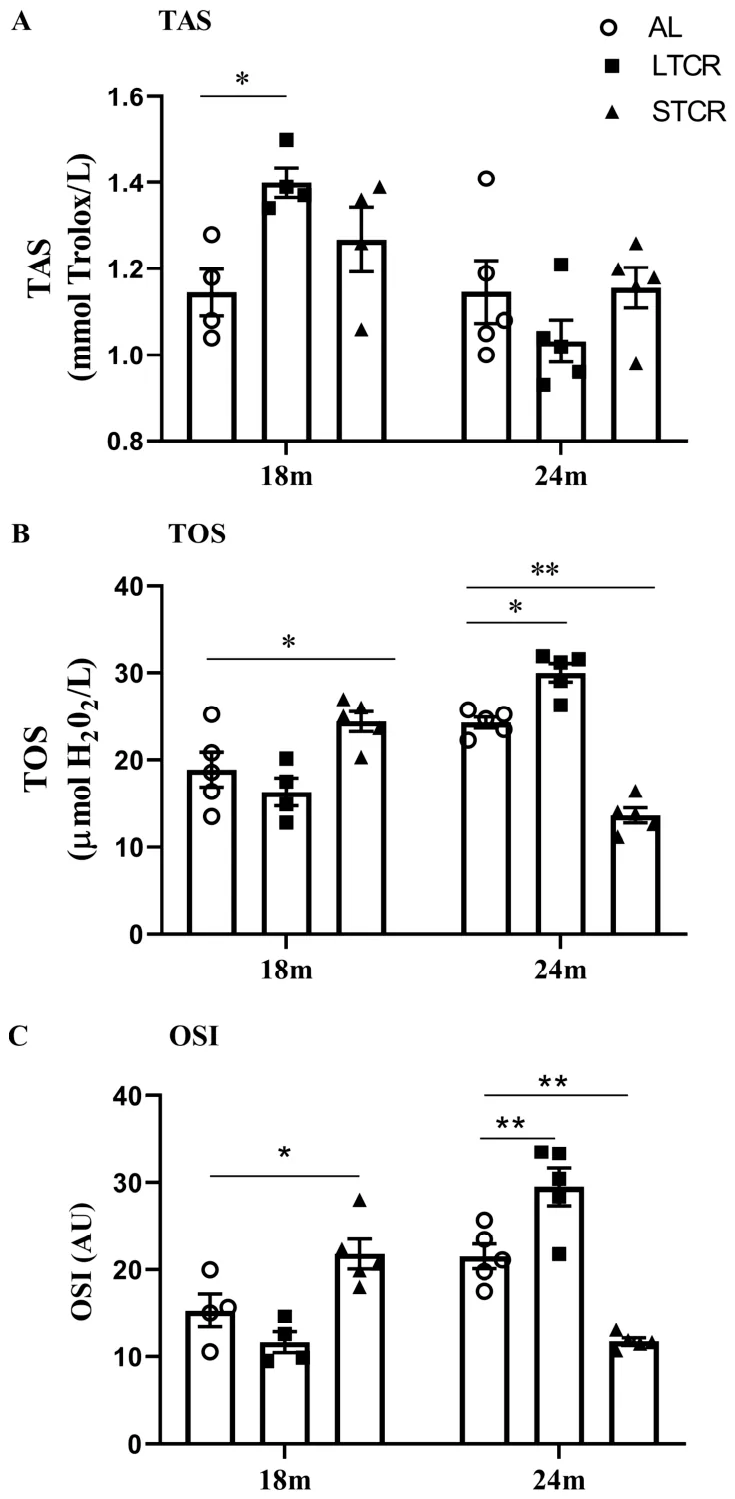
Markers of global redox status. (**A**) Total antioxidant status (TAS); (**B**) total oxidant status (TOS); (**C**) oxidative stress index (OSI). AL, ad libitum; LTCR, long-term caloric restriction; STCR, short-term caloric restriction. Values are presented as mean ± SEM, * *p* < 0.05, ** *p* < 0.01. *n* (18 m AL) = 4–5, *n* (24 m AL) = 5, *n* (18 m LTCR) = 4, *n* (18 m STCR) = 4–5, *n* (24 m LTCR) = 5, *n* (24 m STCR) = 5, One-way ANOVA with Dunnett’s post hoc test.

TAS, representing the cumulative antioxidant capacity of endogenous antioxidants, was not significantly changed in AL-fed animals during aging. It was significantly increased by 27% (*p* < 0.01) only in the 18-month-old group subjected to LTCR (Figure 7A). No changes were observed in the 24-month-old animals after either CR regimen.

TOS, indicating the overall oxidant burden, was influenced by aging and 24-month-old animals have 29% (*p* < 0.05) higher TOS than their 18-month-old peers. In 24-month-old animals, LTCR was associated with significantly increased TOS (by 24%, *p* < 0.01), whereas STCR markedly reduced it by 44% (*p* < 0.001) compared to the AL control (Figure 7B).

OSI, an integrated measure of oxidative stress calculated as the TOS/TAS ratio, was raised (by 40%, *p* < 0.05) in 24-month-old compared to 18-month-old AL-fed animals. There was a trend towards reduction in 18-month-old LTCR animals, while a significant increase (by 42%, *p* < 0.05) was calculated for the 18-month-old STCR group (Figure 7C). In the 24-month-old animals, the OSI was increased by 37% (*p* < 0.05) in the LTCR group, while a reduction by 45% (*p* < 0.01) was detected in the STCR group.

## 3. Discussion

Caloric restriction remains one of the most robust nutritional interventions known to influence aging through modulation of mitochondrial function, energy metabolism, inflammation, and oxidative stress, ultimately promoting maintenance of cellular homeostasis [14,15,25,26,27]. Among organs affected by aging, the brain is particularly vulnerable to oxidative stress because of its high metabolic demand, intensive oxygen consumption, abundance of oxidizable lipids, and relatively limited antioxidant capacity [9]. Consequently, interventions capable of improving redox homeostasis in the brain may have important implications for preserving cognitive function and overall health during aging.

The present study compared CR paradigms differing in onset and duration but terminating at the same age: long-term CR, initiated at 6 months of age and maintained until sacrifice at 18 or 21 months of age, represents a preventive intervention implemented before major age-related alterations become established, while in contrast, short-term CR initiated at either 15 or 21 months of age models late-life intervention introduced when aging-associated physiological decline has already begun. Such paradigms mimic realistic human scenarios, where dietary interventions may be adopted either relatively early in adulthood or much later in life in response to emerging health concerns.

Interpretation of the present findings regarding redox status in the brain should be considered in the context of characterized systemic outcomes in the same animals that were mirrored in the frailty index. Changes in the frailty index provide insight into the overall health status of aging animals, integrating deficits across multiple organ systems. At 18 months of age, both LTCR and STCR improved frailty index compared with age-matched ad-libitum-fed controls, supporting the notion that CR delays age-related deterioration and promotes maintenance of overall physiological function. Notably, some of the strongest contributors to reduced frailty index were cognitive and behavioral components, including spatial working memory (Y-maze) and recognition memory (novel object test). In contrast, no significant improvement in frailty status was observed in the 24-month-old animals. This indicates that the age at which caloric restriction is initiated may be a stronger determinant of outcome than the duration of restriction itself, which is in accordance with our previous findings [20,21]. Earlier studies demonstrated that the period between 18 and 21 months of age in rats represents a critical phase of aging characterized by pronounced morphological and functional alterations in the nervous system, including changes in synaptic protein expression, cholesterol homeostasis, and neuronal plasticity [17,18,28]. Similar observations have been reported by other authors, who suggested that there may be a stage of aging beyond which caloric restriction loses much of its capacity to influence lifespan and healthspan trajectories [29,30,31]. Together, these findings support the hypothesis that successful implementation of CR may depend on intervention timing and that certain age-related alterations become increasingly resistant to modulation once advanced aging is established.

Interestingly, the changes in the frailty index observed in the present experimental paradigm were not fully consistent with those in frailty level assessed by frailty score [19]. Although differences in animal cohorts may partially account for variability in the response to CR, it is evident that frailty scores and the FI exhibit differential sensitivity to CR, as previously observed in female rats [20]. Given that these two frailty assessment tools capture different aspects of multisystem physiological decline, it is not surprising that they do not necessarily classify the same animals as frail [20]. This discrepancy should be carefully considered when frailty measurements are used as outcome measures for evaluating the efficacy of antiaging interventions.

Body weight trajectories in male Wistar rats demonstrated a clear and robust response to 40% caloric restriction, with effects strongly dependent on age at intervention and duration of exposure. While ad-libitum-fed animals exhibited a progressive increase in body weight, reflecting the typical age-associated gain in body mass described in male Wistar rats, long-term CR initiated at 6 months resulted in a rapid reduction in body weight within the first three months, followed by sustained maintenance of this reduced level throughout the study period. At 24 months of age, long-term restricted animals weighed approximately 34% less than age-matched controls, indicating the establishment of a stable low-body-weight phenotype under chronic energy restriction. Similar long-term effects of 40% CR have been reported in rodent models, where early-onset restriction leads to a persistent reduction in body mass of approximately 25–35% compared with ad libitum controls [32].

Short-term CR paradigms on the other hand revealed a distinct age-dependent pattern. When CR was initiated at 15 months, only a modest reduction in body weight was observed (−8.0%), suggesting a relatively attenuated responsiveness at this stage of adulthood. In contrast, CR initiated at 21 months produced a pronounced decrease in body weight (−19.2%), comparable in magnitude to that observed in long-term restricted animals over a similar time window. Notably, late-onset short-term CR did not reduce body weight to the level achieved by long-term restriction, indicating that intervention duration is critical for full metabolic remodeling. These findings are consistent with previous studies on late-onset caloric restriction in rats, which show that, even in advanced age, 40% CR can induce substantial reductions in body weight, although sustained long-term exposure is required to establish a stable low-weight phenotype. For example, studies in aged male rats report body weight reductions of approximately 15–25% following late-life CR, with variability depending on baseline adiposity and duration of restriction [16,32,33].

Importantly, the present findings are also consistent with our previous work in female rats using an identical CR paradigm, where early-onset long-term CR produced a stable reduction in body weight and late-onset CR induced a more variable and less sustained response. Together, these data suggest that, while the magnitude and direction of the body weight response to caloric restriction are broadly conserved across sexes, male Wistar rats may exhibit a more pronounced late-life responsiveness in terms of rapid weight loss, particularly when baseline body weight is elevated. However, females appear to show a more stable adaptation under long-term restriction, indicating potential sex-dependent differences in energy balance regulation and metabolic flexibility. Previous work suggests that lower body weight during adulthood can predict favorable aging outcomes; however, this relationship becomes less straightforward in advanced age, when maintenance of body mass may reflect preserved physiological reserves and resilience [34]. Recent studies have similarly shown that rapid weight loss in aged animals is often associated with reduced physiological robustness, impaired immune competence, and poorer survival [7]. Thus, robust weight loss in the oldest STCR group may reflect reduced adaptive capacity despite evidence of molecular responses to caloric restriction.

The present study extends our previous investigations demonstrating that different CR regimens modulate oxidative stress in the liver and heart, even when initiated late in life [22]. Importantly, the magnitude and direction of these effects differed between organs, supporting the concept of differential aging trajectories and organ-specific responses to oxidative and metabolic stress [3,4,35]. Because the brain possesses unique metabolic characteristics and because cognitive parameters represented major contributors to frailty differences observed in the present cohort, we sought to determine whether alterations in redox homeostasis within the brain could provide mechanistic insight into the functional outcomes associated with CR. This rationale is particularly relevant given that frailty assessment integrates multiple dimensions of health, including cognitive and behavioral performance dependent on intact neuronal function and known to be sensitive to oxidative and metabolic disturbances.

The present study demonstrates that CR modulates brain redox homeostasis in an age-dependent manner, resulting in distinct antioxidant profiles in middle-aged and old animals, although differences in the duration of CR exposure between age groups should be taken into consideration. A plausible explanation for these age-specific responses is provided by the concept of mitochondrial hormesis. Caloric restriction induces a metabolic shift from glucose utilization toward fatty acid oxidation and ketone body production [14,15,16,36,37]. During this transition, temporary increases in mitochondrial ROS generation may occur. Rather than causing damage, moderate oxidative stress could activate adaptive pathways which promote antioxidant defense, mitochondrial maintenance, and cellular repair [38]. While such hormetic responses may improve physiological performance in middle-aged animals with preserved adaptive capacity, the same stimuli in advanced age may primarily activate compensatory mechanisms required to maintain homeostasis. Thus, the stronger glutathione-dependent response observed in old animals may be consistent with preserved redox-regulatory capacity rather than restoration of a younger physiological phenotype [12,39].

In 18-month-old animals, LTCR initiated in early adulthood was associated with increased activities of SOD, CAT, and GST compared with age-matched ad-libitum-fed controls. These enzymes constitute major components of the endogenous antioxidant defense system and play essential roles in detoxifying reactive oxygen species generated during normal metabolism, inflammation, and aging [25]. Interestingly, enhanced enzymatic activity was not accompanied by increased protein expression, suggesting that prolonged CR may regulate antioxidant defenses primarily through post-translational mechanisms, enabling maintenance of antioxidant capacity without the energetic costs of increased protein synthesis. This represents an efficient adaptation to prolonged energy restriction and is consistent with the concept that CR promotes metabolic optimization rather than simply increasing antioxidant abundance.

Although LTCR enhanced antioxidant enzyme activities, it did not significantly reduce lipid peroxidation or protein carbonylation, which may appear contradictory. However, enhanced antioxidant activity may precede measurable reductions in accumulated oxidative damage and instead reflect improved resilience of neuronal tissue. The observation that LTCR animals simultaneously exhibited improved frailty outcomes supports the interpretation that this antioxidant phenotype is biologically meaningful and likely contributes to maintenance of physiological function during aging.

A somewhat different pattern was observed following STCR in 18-month-old animals. In addition to increased CAT activity, elevated expression of SOD and iNOS proteins was detected. Such findings could suggest activation of adaptive stress-response pathways triggered by the relatively recent implementation of CR. The increase in iNOS expression may initially seem to be controversial. Namely, iNOS is commonly associated with neuroinflammatory processes and increased nitric oxide production under pathological conditions [24]. However, moderate induction of iNOS is frequently observed during hormetic adaptation and may reflect activation of cellular signaling pathways in stress conditions rather than pathological inflammation [40]. In the absence of direct assessment of inflammatory markers, the present data do not allow discrimination between these possibilities. Together, the molecular profile of STCR animals seems consistent with an active adaptive response to metabolic challenge. Importantly, as with LTCR, these molecular changes occurred alongside improved frailty outcomes, indicating that activation of antioxidant defenses in middle-aged animals may be associated with functional benefits at the organismal level.

Therefore, despite differences in the specific molecular pathways activated by long- and short-term restriction, both interventions in 18-month-old animals likely promote a beneficial adaptive phenotype characterized by enhanced redox resilience and improved physiological status. LTCR may represent a stable, established adaptation, whereas STCR may reflect an earlier stage of the same adaptive process.

The response observed in 24-month-old animals was markedly different. In this age group, STCR was characterized by pronounced activation of glutathione-dependent antioxidant pathways, including increased activities of GSH-Px and GR. These changes were accompanied by reductions in lipid peroxidation and protein oxidation as well as lower TOS and OSI values, indicating improved oxidative balance. Given the central role of the glutathione system in detoxifying hydrogen peroxide and organic hydroperoxides [25,37], increased GSH-Px activity likely contributed directly to the observed reduction in oxidative damage. Nevertheless, interpretation of these findings requires caution. Unlike the 18-month-old animals, improvements in redox markers were not paralleled by improvements in frailty. Indeed, frailty outcomes remained unfavorable despite activation of antioxidant defenses and reduction of oxidative damage. This discrepancy suggests that the biological meaning of antioxidant activation may differ in advanced age. Rather than reflecting enhanced physiological health, the increased activity of glutathione-related enzymes may also be interpreted as a compensatory response aimed at maintaining redox homeostasis in the context of age-related dysfunction. Similar patterns of upregulation of protective systems have been reported in aging, although such responses do not necessarily indicate improved physiological function [25,41].

Our previous findings in the same animal model suggest that the biological effects of CR are complex and may depend on age, tissue, and the physiological context [16,19,21]. Short-term CR in aged animals improved some markers of oxidative stress in liver and heart [22] and restored age-related imbalance of lipid metabolism [16] but also had negative impacts on hepatic inflammatory status, behavioral outcomes and frailty level. Thus, we have shown that CR-associated molecular improvements in several tissues do not always translate into proportional improvements in physiological condition and a similar discrepancy was shown by others [42]. Such discrepancies highlight an important limitation of relying exclusively on molecular biomarkers when evaluating interventions targeting aging. Although oxidative stress markers provide valuable mechanistic information, they do not necessarily predict organismal health or functional performance. The present findings reinforce the concept that successful aging should be assessed through integration of molecular, physiological, and functional endpoints. The results also support the concept of differential aging trajectories. In the same animals, CR-induced changes in heart and liver tissue were primarily characterized by alterations in CAT activity [22], whereas the brain exhibited a considerably more complex response involving enzymatic and non-enzymatic antioxidant systems. Such organ-specific responses are consistent with growing evidence that individual tissues age at different rates and utilize distinct adaptive mechanisms [4,35]. Consequently, interventions that appear beneficial at the molecular level in one tissue may have neutral or even adverse consequences in another [31]. It should also be considered that oxidative stress may not solely represent a causal driver of functional decline but may also arise as a consequence of age-related physiological deterioration [38]. Therefore, the observed redox alterations should be interpreted within the broader context of aging-related changes rather than as direct indicators of causality.

While our data provide evidence of CR-related modulation of redox homeostasis, some limitations must be noted. First, as analyses were conducted on whole-hemisphere homogenates to assess global brain alterations associated with aging, region-specific differences may be masked [36]. In particular, the hippocampus, which is known for its heightened vulnerability to aging, may exhibit more pronounced changes that cannot be resolved using this method. Future studies employing region-specific analyses will be necessary to better define the spatial specificity of the observed effects. Second, comparisons between the 18-month-old and 24-month-old groups, as well as comparisons within each age group, reflect differences not only in chronological age but also in the duration of caloric restriction exposure. Therefore, we recognize that the observed differences likely reflect condition-specific physiological states at the time of analysis and should not be overinterpreted as direct causal effects of caloric restriction per se. Because the timing and duration of dietary interventions differ across groups, the design does not support a balanced factorial framework, and thus interaction effects between age and diet (i.e., two-way ANOVA) cannot be reliably assessed. Further, mitochondrial markers and transcriptional regulators (e.g., PGC-1α, Nrf2, SIRT1, FOXO3, …) were not assessed directly as the present study was designed to characterize redox outcomes rather than upstream signaling pathways; therefore, proposed mechanisms remain speculative and need future analyses to better elucidate mechanistic pathways at specific ages [14,15,37]. It should also be noted that animals consumed their daily ration within a relatively short time window (approximately 3 h), indicating that the applied caloric restriction paradigm may have also incorporated elements of intermittent fasting or time-restricted feeding. As such, the observed effects cannot be attributed exclusively to reduced caloric intake, since the timing and rhythm of feeding may have contributed. Disentangling the specific effects of caloric restriction and fasting-related mechanisms would require experimental designs that independently control energy intake and feeding patterns.

Altogether, the present findings indicate that the aging brain retains the ability to mount differential antioxidant responses even late in life, but the biological significance of this response may change across the aging trajectory. In middle-aged animals, activation of antioxidant defenses by both long- and short-term CR was associated with improved frailty outcomes, which may reflect a beneficial adaptive response that enhances resilience to age-related stress. In contrast, the pronounced glutathione-dependent response observed in old animals may be more consistent with a compensatory mechanism aimed at maintaining redox homeostasis in the presence of advanced physiological decline. While these observations are compatible with a model in which caloric restriction engages distinct redox-related responses depending on age, the present data do not allow direct assessment of redox plasticity as a dynamic adaptive capacity. Further studies specifically designed to assess functional redox adaptability would be required as that may be important for interpreting aging-related biomarkers and optimizing dietary interventions to promote healthy aging.

## 4. Material and Methods

### 4.1. Animals

Adult male Wistar rats (*Rattus norvegicus*) were obtained from the breeding colony of the Institute for Biological Research “Sinisa Stankovic”—National Institute of the Republic of Serbia, University of Belgrade (Belgrade, Serbia). Animals were housed under standard laboratory conditions: constant temperature (21 ± 2 °C), controlled humidity (55 ± 10%), and a 12 h light/12 h dark cycle. Standard rat chow (Table 1) and water were provided ad libitum.

All experimental procedures were conducted in accordance with European Directive 2010/63/EU and the Directive 86/609/EEC of the Council of the European Communities on the protection of animals used for scientific purposes. The study protocol was approved by the Ethics Committee for the Use of Laboratory Animals of the Institute and by the National Research Ethics Committee (#323-07-13536/2020-05, approved on 23 December 2020).

### 4.2. Experimental Design

The animals were initially divided into two groups: ad-libitum-fed (AL, control) and calorie-restricted (CR). The AL group had unrestricted access to food until 18 months (late-middle-aged) or 24 months (aged) of age. The CR groups received 60% of the average daily food intake of the AL group, corresponding to 40% calorie restriction. Before initiation of calorie restriction, food intake of the animals assigned to the CR groups was measured at the cage level over a 10-day period by recording the amount of food provided and the remaining food after 24 h. Average daily food intake per rat was calculated by dividing the total cage intake by the two rats housed in each cage. This average intake was then used to calculate the daily food allowance corresponding to 60% of AL intake, and the resulting amount was provided as the total daily ration for each cage. No detectable food spillage was observed during these measurements; therefore, no correction for food wastage was required. Food intake was reassessed at 15, 18, and 21 months of age to account for age-related changes in consumption, and the CR ration was adjusted accordingly to maintain the same level of restriction. To facilitate adaptation to the food restriction, CR animals were gradually acclimated: food amount was reduced by 10% every two days until reaching the target of 60% of AL intake within seven days [21]. CR rats received their measured daily food ration at 15:00. All animals had free access to water. Body weight was measured daily during the first month of calorie restriction to monitor adaptation to the dietary regimen and detect excessive weight loss or signs of malnutrition. Thereafter, body weight was recorded once weekly until the end of the experiment.

**Scheme 1:**
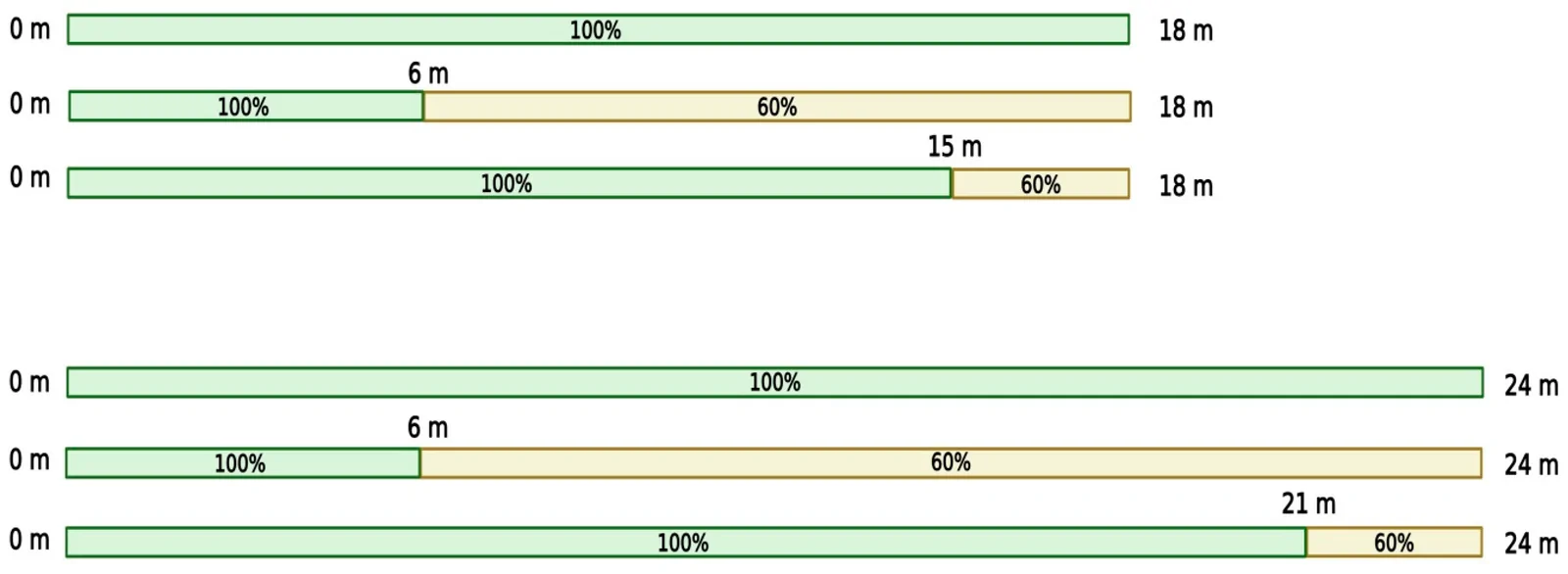
Timing and duration of caloric restriction in aging rats. Long-term CR (LTCR) was initiated at 6 months of age and continued until either 18 months (18-month LTCR) or 24 months of age (24-month LTCR,). Short-term CR (STCR) was applied from 15 to 18 months (18-month STCR) or from 21 to 24 months of age (24-month STCR). Age-matched control groups were maintained on ad libitum (AL) feeding until 18 months or 24 months of age. All animals were sacrificed at either 18 or 24 months.

At 6 months of age, the CR group was further subdivided according to the duration and onset of restriction. The long-term calorie restriction (LTCR) group underwent restriction from 6 months of age until sacrifice at 18 or 24 months. The short-term calorie restriction (STCR) group underwent restriction for 3 months, starting at either 15 or 21 months of age until sacrifice at 18 and 24 months, respectively (Scheme 1).

The wellbeing of the rats was monitored carefully throughout the experiment. At 18 and 24 months of age, animals were sacrificed. Anesthesia was induced by intraperitoneal injection of Zoletil (Virbac, Carros, France, 50 mg/kg body weight). Rats were perfused transcardially with 0.9% NaCl, after which brains were quickly removed and stored at −80 °C until analysis. One hemisphere of each brain was designated for biochemical and oxidative stress assays.

Sample size was determined based on our long-standing experience with this experimental model and similar biochemical and behavioral analyses, considering the variability and effect sizes observed in previous studies. A formal a priori sample size calculation was not performed. Animal numbers were kept to the minimum necessary to obtain reliable data, in accordance with the 3Rs (Replacement, Reduction, and Refinement), while also taking into account resource and animal availability. Animals exhibiting severe illness, persistent inability to access food or water, marked weight loss beyond that expected from the dietary intervention, or other signs of significant distress would have been humanely euthanized before the planned experimental endpoint.

Animals were assigned to experimental groups based on the predefined study design (age and dietary intervention). Within each age cohort, animals were allocated using a body-weight-balanced procedure to ensure comparable baseline characteristics between groups. Blinding during animal handling, behavioral experiments and sample collection was not feasible because the experimental groups differed in a chronological age and the dietary intervention itself made calorie-restricted animals readily identifiable by their lower body weight, making group allocation apparent. However, tissue samples were coded, and biochemical quantification and statistical analyses were performed with the investigator blinded to group allocation. In addition, frailty scoring was performed by an investigator blinded to group allocation (to the extent possible, as age and body weight differences were obvious).

### 4.3. Determination of Frailty Index

Calculation of a frailty index was based on our previous work [21]. We included 23 parameters in our scale, 21 regarding condition of different organ systems, and discrimination index from a novel object test (DI) and spontaneous activity from a Y maze test (SAB) as the 22nd and 23rd parameters, respectively (Supplementary Table S1). Animals (total *n* = 69) were allocated to the following experimental groups according to the study design (no formal randomization was used): 6-month AL (*n* = 11), 18-month AL (*n* = 10), 24-month AL (*n* = 14), 18-month LTCR (*n* = 9), 18-month STCR (*n* = 8), 24-month LTCR (*n* = 8), and 24-month STCR (*n* = 9). Each animal was assessed in a brief clinical examination and the severity of each deficit was rated with a simple scale: 0 (no sign of a deficit), 0.5 (mild deficit), and 1 (severe deficit). Having in mind that, among studies, the lower point of the DI value indicating novelty preference usually varies from 0.2–0.5, we created the following scale for FI: DI value of 0.5–1 was scored as 0, value of 0.2–0.5 was scored 0.5 and value ≤ 0.2 was scored as 1 (25) (Supplementary Table S2). For SAB, SAB values higher than 50% chance were scored as 0, SAB values that were lower than 50% were scored 0.5 and animals that had fewer than 9 entries during the experiment were scored 1. All these values were then summed, and the total was divided by the number of parameters measured to provide a frailty index score between 0 and 1 for each animal. To reduce potential confounding associated with the order of measurements, the sequence of behavioral assessments was manually alternated among the experimental groups throughout the study, ensuring that no group was consistently tested first or last. Since weight loss is an expected consequence of dietary restriction, body weight was excluded from the FI calculation and analyzed separately. Analysis of FI was performed using ordinary one-way ANOVA followed by Dunnett’s multiple-comparison post hoc test for the effects of aging and different feeding regimens. For evaluation of alternation above chance, a one-sample *t*-test was used.

### 4.4. Determination of Oxidative Stress Biomarkers in the Brain

#### 4.4.1. Tissue Preparation

Biochemical analyses were performed on one brain hemisphere, encompassing multiple regions including the cerebral cortex, hippocampus, and striatum. This approach was selected to provide an integrated assessment of global brain alterations associated with aging, as these regions are all known to be affected, albeit to different extents.

Oxidative stress biomarkers were analyzed in a randomly selected subset of animals from each experimental group: 18-month AL (*n* = 5), 24-month AL (*n* = 5), 18-month LTCR (*n* = 5), 24-month LTCR (*n* = 5), 18-month STCR (*n* = 5), and 24-month STCR (*n* = 5), making total *n* = 30.

Brain tissue samples were minced and homogenized on ice in 10 volumes of 25 mmol/L sucrose containing 5 mmol/L Tris-HCl (pH 7.5) and 0.1 M EDTA, supplemented with 1× phosphatase inhibitor mix I and 1× protease inhibitor mix G, at 4 °C [43], using an IKA-Werk Ultra-Turrax homogenizer (Janke and Kunkel, Staufen, Germany) [44]. Homogenates were then sonicated on ice at 10 kHz for 30 s (Bandeline Sonopuls, Berlin, Germany) to release enzymes [45], followed by ultracentrifugation at 100,000× *g* for 90 min at 4 °C using a Beckman (Beckman Coulter Life Sciences, Indianapolis, IN, USA) ultracentrifuge [46,47].

#### 4.4.2. Antioxidant Enzyme Assays

The activity of superoxide dismutase (EC 1.15.1.1) was determined by the epinephrine method [48], based on the capacity of SOD to inhibit the autoxidation of adrenaline to adrenochrome. One unit of SOD activity was defined as the amount of protein causing 50% inhibition of the autoxidation of adrenaline at 37 °C and was expressed as total activity in U/g wet mass.

Catalase (EC 1.11.1.6) activity was evaluated by the rate of hydrogen peroxide (H_2_O_2_) decomposition [49]. The method is based on H_2_O_2_ degradation by the action of CAT contained in the examined samples. In this procedure 50 mM phosphate buffer (pH 7.0) was used and 30 mM H_2_O_2_ as substrate. CAT activity was expressed as μmol H_2_O_2_/min/g wet mass.

The activity of glutathione peroxidase (EC 1.11.1.9) was determined following the oxidation of nicotinamide adenine dinucleotide phosphate (NADPH) with t-butyl hydroperoxide as a substrate [50]. This reaction is followed by the action of GSH-Px contained in the samples examined on t-butyl hydroperoxide (3 mM) as substrate in 0.5 M phosphate buffer, pH 7.0, at 37 °C. The activity of GSH-Px was expressed as nmol NADPH/min/g wet tissue.

Glutathione reductase (EC 1.6.4.2) activity was measured using the method of Glatzle et al. [51]. The method is based on the capability of GR to catalyze the reduction of oxidized glutathione (GSSG) to reduce glutathione (GSH) using NADPH as substrate in the phosphate buffer (pH 7.4). GR activity was expressed as nmol NADPH/min/g wet tissue.

The activity of glutathione S-transferase (EC 2.5.1.18) towards 1-chloro-2,4-dinitrobenzene (CDNB) was determined by the method of Habig et al. [52]. The method is based on the reaction of CDNB with the -SH group of GSH catalyzed by GST contained in the samples. The reaction proceeded in the presence of 1 mM GSH in phosphate buffer (pH 6.5) at 37 °C. GST activity was expressed as nmol GSH/min/mg protein and as nmol GSH/min/g wet tissue.

All enzyme assays were performed in triplicate using a Shimadzu UV-1900i spectrophotometer (Shimadzu Corporation, Kyoto, Japan) equipped with a temperature-controlled cuvette holder maintained at 37 °C. Mean values from the three independent measurements were used for statistical analysis. All reagents were purchased from Sigma-Aldrich (St. Louis, MO, USA).

#### 4.4.3. Determination of Lipid Peroxidation and Protein Carbonylation

Lipid peroxidation was assessed by measuring thiobarbituric acid reactive substances (TBARSs) according to the method of Rehncrona et al. [53]. In this assay, thiobarbituric acid (TBA) reacts with lipid peroxides to form a colored complex, the absorbance of which was measured spectrophotometrically at 532 nm. TBARS concentrations were calculated using the fixed molar extinction coefficient for the MDA–TBA complex (ε = 1.97 × 10^6^ M^−1^ cm^−1^). Results were expressed as nmol TBARS/mg tissue.

Protein carbonylation (PCO) was determined using the method of Mesquita et al. [54]. This assay is based on the reaction of 2,4-dinitrophenylhydrazine (DNPH) with protein carbonyl groups (C=O), forming 2,4-dinitrophenylhydrazone, which exhibits a maximum absorbance at 450 nm under alkaline conditions. Results were expressed as nmol/g wet mass.

#### 4.4.4. Determination of TOS, TAS and OSI

Total oxidant status (TOS) and total antioxidant status (TAS) were measured using commercial Colorimetric Assay Kits (Elabscience, Houston, TX, USA), following the manufacturer’s instructions (Catalog Nos. E-BC-K802-M and E-BC-K801-M).

The principle of the TOS assay is based on the oxidation of Fe^2+^ to Fe^3+^ by oxidizing agents present in the sample under acidic conditions. Fe^3+^ subsequently forms a stable blue-purple complex with xylenol orange, which is measured spectrophotometrically at 590 nm using a TAC-SYNERGY H1 Microplate Reader (BioTek, Agilent, CA, USA). The intensity of the color is directly proportional to the total oxidant content. Results were expressed as μmol H_2_O_2_ equivalents per gram of wet tissue.

The TAS assay is based on the ability of antioxidants present in the sample to reduce the 2,2′-azino-bis (3-ethylbenzothiazoline-6-sulfonic acid) (ABTS) radical cation, which is generated by the oxidation of ABTS with an appropriate oxidizing agent. The reduction of ABTS causes decolorization, which is quantified by measuring absorbance at 660 nm using the same microplate reader. Trolox was used as the reference standard, and the results were expressed as mmol Trolox equivalents per gram of wet tissue.

The oxidative stress index (OSI) represents the deviation from the physiological oxidative balance, where a theoretical value of zero indicates perfect equilibrium between pro-oxidant and antioxidant components. OSI was calculated as (TOS/TAS) × 100 ratio. Lower OSI values reflect a redox state closer to balance, whereas higher values indicate increasing oxidative imbalance. Elevation of the OSI may result from either an increase in pro-oxidant species or a reduction in antioxidant capacity. OSI values were expressed in arbitrary units (AU) [24].

### 4.5. Western Blot Analysis

Brain tissue was homogenized and sonicated in 10 volumes of RIPA buffer (50 mM Tris-HCl, pH 7.5; 150 mM NaCl; 1% NP-40; 0.5% Triton X-100; 0.1% SDS; 1 mM EDTA; 1 mM EGTA) supplemented with a complete protease and phosphatase inhibitor cocktail (Roche, Mannheim, Germany). Homogenates were centrifuged at 16,000× *g*, 4 °C, and the resulting supernatants were collected and stored at −80 °C until analysis. Protein concentrations were determined using the Micro BCA^TM^ Protein Assay Kit (Thermo Fisher Scientific Inc., Waltham, MA, USA) with bovine serum albumin (BSA) as a standard.

Equal amounts of protein (15 μg per lane) were separated on 10% SDS-PAGE gels and transferred to Amersham™ Protran 0.45 NC nitrocellulose membranes (Cytiva, /Global Life Sciences Solutions, Marlborough, MA, USA). Membranes were briefly stained with 1% Ponceau S in 5% glacial acetic acid to verify protein transfer. Blocking was performed at room temperature for 1 h in either 5% non-fat dry milk (Santa Cruz Biotechnology, Inc., Dallas, TX, USA) or 3% BSA (Merck KGaA, Darmstadt, Germany) prepared in TBST buffer (50 mM Tris, pH 7.0; 150 mM NaCl; 0.05% Tween 20).

Membranes were incubated overnight at 4 °C with primary antibodies against SOD1 (SCTB, sc271014, 1:1000), CAT (SCTB, sc271803, 1:1000), and iNOS (Abcam, Cambridge, UK, ab15323, 1:1000), diluted in blocking buffer. After washing in TBST, membranes were incubated for 1 h at room temperature with HRP-conjugated secondary antibodies (rabbit anti-mouse, Dako P0260, 1:3000 and goat anti-rabbit, Abcam, Cambridge, United Kingdom, ab205718, 1:5000). Protein bands were visualized using chemiluminescence on an iBright imaging system (Thermo Fisher Scientific Inc., Waltham, MA, USA). Signal intensities were quantified with ImageQuant software (v. 5.2; GE Healthcare, Chicago, IL, USA). Each target protein signal was normalized to the signal obtained from a corresponding membrane stained with a Pierce Reversible Protein Stain Kit (Thermo Fisher Scientific, Waltham, MA, USA). Total membrane staining was used as a loading control, as this type of signal normalization is considered a robust and reliable approach for assessing equal loading and transfer efficiency [55]. To ensure accurate quantification, the linearity of total protein signal with respect to protein loading was verified in preliminary experiments under the same electrophoresis, transfer, and staining conditions. A pooled protein sample derived from study animals was loaded across a range of concentrations (10–30 µg), confirming that the amount used in this study (15 µg) fell within the linear dynamic range. For inter-membrane comparisons, a pooled protein sample was used as an internal reference. Protein levels were expressed as actual densitometric values normalized to total protein load fold change.

### 4.6. Statistical Analysis

Statistical analyses were performed using GraphPad Prism 8 software (GraphPad Software Inc., La Jolla, CA, USA). Normality of data distribution was assessed using the D’Agostino–Pearson test. Comparisons between the groups were performed using one-way ANOVA followed by Dunnett’s post hoc test for multiple comparisons against the corresponding age-matched AL control group. To determine age-related changes between 18- and 24 month-old ad-libitum-fed animals Student’s *t*-test was used. Data are presented as mean ± standard error of the mean (SEM). Individual data points represent values obtained from individual animals. The level of statistical significance was set at *p* < 0.05. Potential outliers were identified using the GraphPad Prism ROUT method (Q = 1%) and excluded according to the predefined criterion.

## Figures and Tables

**Table 1:** Composition of the standard rat food used in the experiment.

**Chemical Composition of Rat Diet**		
**General**	**Vitamins**	**Minerals**
Proteins min 20.00% Fat min 5.00% Carbohydrates max 49% Humidity max 13.5% Cellulose max 5.00% Ashes max 8.00% Calcium min 0.95% Phosphorus min 0.70% Lysine min 0.60% Methionine min 0.60% Tryptophan max 0.15%	Vit A min 12.000 U/kg Vit D3 min 2.000 U/kg Vit E min 30.00 mg/kg Vit B1 min 4.00 mg/kg Vit B2 min 7.00 mg/kg Vit B6 min 6 mg/kg Vit B12 min 0.05 mg/kg Folic acid min 1.0 mg/kg Choline chloride 1.000.00 mg/kg Niacin 20.00 mg/kg Pantothenic acid min 10.00 mg/kg	Zinc min 30 mg/kg Copper min 15.00 mg/kg Iron min 60 mg/kg Manganese min 60 mg/kg Iodine min 0.15 mg/kg Selenium min 0.10 mg/kg
		Antioxidants E-321, E-320, E-338, E-202 min 125 mg/kg

## Data Availability

The raw data supporting the conclusions of this article will be made available by the authors on request.

## Supplementary Materials

The following supporting information can be downloaded at: https://www.mdpi.com/article/10.3390/ijms27167229/s1.
