# Supplementary material for: Duration of Caloric Restriction Modulates Brain Redox Adaptation and Frailty During Aging
Source: Int J Mol Sci. 2026 Aug 13;27(16):7229. doi: 10.3390/ijms27167229 (PMC13513332; doi:10.3390/ijms27167229)
Supplement: Supplementary file 1 [file ijms-27-07229-s001.zip › ijms-4444247-supplementary.pdf]

Supplementary Table S1. Clinical assessment of deficits in aging AL and CR male rats to create a frailty index.

System/ Parameter	Clinical assessment of deficit	Scoring
**Integument**		
Alopecia	Gently restrain the animal and inspect it for signs of fur loss.	0 = normal fur density<br>0.5 = < 25% fur loss<br>1 = >25% fur loss
Dermatitis	Document skin lesions.	0 = absent<br>0.5 = focal lesions (*e.g.* neck, flanks, under chin)<br>1 = widespread or multifocal lesions
Coat condition	Inspect the animal for signs of poor grooming.	0 = smooth, sleek, shiny coat<br>0.5 = coat is slightly ruffled<br>1 = unkept and un-groomed, matted appearance
**Physical/ Musculoskeletal**		
Tumours	Observe the animal to look for symmetry. Hold the base of the tail and manually examine the rat for visible or palpable tumours.	0 = absent<br>0.5 = <1.0 cm<br>1 = >1.0 cm or multiple smaller tumours
Distended abdomen	Hold the animal vertically by the base of their tail and tip backwards over your hand. Excess fluid visible as a bulge below the rib cage.	0 = absent<br>0.5 = slight bulge<br>1 = abdomen clearly distended
Tremor	Observe the freely moving animal to detect tremor, both at rest and when the animal is trying to climb up an incline.	0 = no tremor<br>0.5 = slight tremor<br>1 = marked tremor; animal cannot climb
Body condition	Place the animal on a flat surface, hold tail base and manually assess the flesh/fat that covers the sacroiliac region (back and pubic	0 = bones palpable, not prominent<br>0.5 = bones prominent or barely felt<br>1 = bones very prominent or not felt due

score	bones).	to obesity
**Vestibulocochlear/ Auditory**		
Hearing loss	Test startle reflex. Clap hands and record responses. Repeat 3 times. Make sure that the animal is facing the opposite side from experimentator.	0 = always reacts (3/3 times)<br>0.5 = reacts 1/3 or 2/3 times<br>1 = unresponsive (0/3 times)
**Ocular/nasal**		
Cataracts	Visual inspection of the animal to detect opacity in the centre of the eye.	0 = no cataract<br>0.5 = small opaque spot<br>1 = clear evidence of opaque lens
Microphthalmos	Inspect eyes.	0 = normal size<br>0.5 = one or both eyes slightly small or sunken<br>1 = one or both eyes very small or sunken
Exophthalmos	Inspect eyes.	0 = normal size<br>0.5 = one or both eyes slightly bigger or bulging<br>1 = one or both eyes very big or bulging
**Neurological**		
Head tilt	Inspect the animal's acitivity for a sign of a head tilt.	0 = no head tilt<br>0.5 = slight head tilt in one side<br>1 = severe head tilt, loss of balance and rolling
**Digestive/Urogenital**		
Rectal prolapse	Grasp the animal by the base of the tail to detect signs of rectal prolapse.	0 = no prolapse<br>0.5 = small amount of rectum visible below tail

		1 = rectum clearly visible below tail.
Penile prolapse	Grasp the animal by the base of the tail to detect signs of vaginal/uterine prolapse.	0 = no prolapsed<br><br>0.5 = small amount of prolapsed tissue visible<br><br>1 = prolapsed tissue clearly visible
Diarrhoea	Grasp the animal and invert it to check for signs of diarrhoea. Also look for fecal smearing in the home cage.	0 = none<br><br>0.5 = some feces or bedding near rectum<br><br>1 = feces plus blood and bedding near rectum, home cage smearing
Jaundice	Examine animals for the presence of yellow colour of sole of feet, nose, ears and tail.	0 = no yelow staining<br><br>0.5 = yelow staining in traces<br><br>1 = intensive yelow staining
**Respiratory**		
Breathing rate/depth	Observe the animal. Note the rate and depth of breathing as well as any gasping behaviour.	0 = normal<br><br>0.5 = modest change in breathing rate and/or depth<br><br>1 = marked changes in rate/depth, gasping
Unusual sounds	Examine the animal for unusual breathing sounds as a symptom of upper and lower respiratory impairment, such as wheezing and rattling.	0 = no unusual sound<br><br>0.5 = occasional and quiet breathing sound<br><br>1 = constant or frequent sound of wheezing and rattling
**Other**		
Hunched posture	Examine the animal for „head down feet together" posture as a sign of pain or disease.	0 = no hunched posture<br><br>0.5 = slight hunched posture<br><br>1 = severe hunched posture
Chromodacryorrhea	Examine rats for porphyrin staining around eyes and nose.	0 = no porphyrin staining<br><br>0.5 = porphyrin staining in traces<br><br>1 = intensive porphyrin staining

Malocclusion	Examine rats for incisors growth.	0 = no incisors growth<br><br>0.5 = moderate incisors growth<br><br>1 = severe incisors growth
Discrimination index	Calculate novel object discrimination using formula: t novel-t familiar) / (t novel+ t familiar).	0 = Discrimination index value of 0.5-1<br><br>0.5 = Discrimination index value of 0.2-0.5<br><br>1 = Discrimination index value ≤0.2
Spontaneous alternations	Calculate spontaneous alternation.	0 = more than total of 9 entries and alternation at levels significantly above chance (50%)<br><br>0.5 = Animals did not alternated at levels significantly above chance (50%).<br><br>1 = Less than total of 9 entries in the arms during experiment

Supplementary Table S2. Individual frailty parameters scores used to develop a clinical frailty index in AL male rats during aging.

	Experimental group	6 months AL (n=11)			18 months AL (n=10)			24 months AL (n=14)		
	**Score**	**0**	**0.5**	**1**	**0**	**0.5**	**1**	**0**	**0.5**	**1**
**Parameters**[1]	Alopecia	11	0	0	10	0	0	14	0	0
	Dermatitis	11	0	0	10	0	0	14	0	0
	Coat condition	10	1	0	4	6	0	8	6	0
	Tumors	11	0	0	10	0	0	7	1	6
	Tremor	11	0	0	9	1	0	14	0	0
	Distended abdomen	11	0	0	10	0	0	14	0	0
	Body condition score	9	2	0	9	1	0	13	0	0
	Hearing loss	10	1	0	9	1	0	8	0	6
	Head tilt	11	0	0	10	0	0	14	0	0
	Cataracts	11	0	0	10	0	0	14	0	0
	Exophthalmos	11	0	0	9	0	1	11	1	2
	Microphthalmos	11	0	0	9	0	1	14	0	0
	Diarrhoea	11	0	0	9	0	1	13	1	0
	Jaundice	11	0	0	10	0	0	14	0	0
	Penile prolapse	11	0	0	10	0	0	13	1	0
	Rectal prolapse	10	1	0	6	1	3	10	2	2
	Breathing rate/depth	10	1	0	9	0	1	12	0	2
	Unusal sounds	11	0	0	10	0	0	14	0	0
	Hunched posture	11	0	0	10	0	0	13	1	0
	Chromodacryorrhea	9	0	2	9	0	1	12	0	2
	Malocclusion	11	0	0	10	0	0	13	0	1
	Discrimination index (DI)	6	5	0	3	3	4	5	7	2
	Spontaneous alternations	6	5	0	0	6	4	8	1	5

	(SAB)									

[1]Values represent the number of rats with frailty scores of 0, 0.5 or 1 for each parameter used to develop a frailty index. AL-ad libitum

Supplementary Table S3. Individual frailty parameters scores used to develop a clinical frailty index in LTCR and STCR male rats.

	Experimental group	18 months LTCR (n=9)			18 months STCR (n=8)			24 months LTCR (n=8)			24 months STCR (n=9)		
	**Score**	**0**	**0.5**	**1**	**0**	**0.5**	**1**	**0**	**0.5**	**1**	**0**	**0.5**	**1**
**Parameters[1]**	Alopecia	9	0	0	8	0	0	8	0	0	9	0	0
	Dermatitis	9	0	0	8	0	0	8	0	0	9	0	0
	Coat condition	6	2	1	6	2	0	5	3	0	6	3	0
	Tumors	7	0	2	7	1	0	4	0	4	8	1	0
	Tremor	9	0	0	8	0	0	8	0	0	9	0	0
	Distended abdomen	9	0	0	8	0	0	8	0	0	9	0	0
	Body condition score	7	2	0	6	2	0	7	0	1	8	1	0
	Hearing loss	7	2	0	6	1	1	4	2	2	4	1	4
	Head tilt	9	0	0	8	0	0	8	0	0	9	0	0
	Cataracts	9	0	0	8	0	0	8	0	0	9	0	0
	Exophthalmos	9	0	0	7	1	0	8	0	0	8	1	0
	Microphthalmos	9	0	0	8	0	0	7	0	1	9	0	0
	Diarrhoea	9	0	0	8	0	0	8	0	0	9	0	0
	Jaundice	9	0	0	8	0	0	8	0	0	8	0	1
	Penile prolapse	9	0	0	8	0	0	8	0	0	9	0	0
	Rectal prolapse	8	1	0	6	2	0	6	2	0	6	3	0
	Breathing rate/depth	8	1	0	7	1	0	6	2	0	7	1	1
	Unusal sounds	9	0	0	8	0	0	8	0	0	9	0	0
	Hunched posture	9	0	0	8	0	0	8	0	0	9	0	0
	Chromodacryorrhea	9	0	0	8	0	0	8	0	0	8	1	0
	Malocclusion	9	0	0	8	0	0	8	0	0	9	0	0
	Discrimination index (DI)	1	5	3	2	3	3	0	2	6	2	3	4

	Spontaneous alternations (SAB)	5	3	1	6	2	0	4	3	1	2	4	3

[1]Values represent the number of rats with frailty scores of 0, 0.5 or 1 for each parameter used to develop a frailty index. LTCR-long-term caloric restriction; STCR-short-term caloric restriction.
